# Bacteriological Profile of Nosocomial Pneumonia and Current State of Antibiotic Resistance in the Military Hospital of Avicenne

**DOI:** 10.7759/cureus.68125

**Published:** 2024-08-29

**Authors:** Mehdi Didi, Said Khallikane, Youssef Qamouss, Lamiae Arsalane, Said Zouhair

**Affiliations:** 1 Anesthesiology and Reanimation, Military Hospital of Avicenne, Marrakech, MAR; 2 Microbiology, Military Hospital of Avicenne, Marrakech, MAR

**Keywords:** resistance, pneumonia, nosocomial, bacteria, antibiotics

## Abstract

This retrospective study, conducted over five years, aimed to assess the bacteriological profile of nosocomial pneumonia, the antibiotic resistance of isolated bacteria, and changes in these parameters over time. The analysis reviewed 660 samples from the microbiology department at the Military Hospital of Avicenne in Marrakech, Morocco, covering the period from January 1, 2017, to December 31, 2021. Among these samples, 303 microorganisms were identified from 251 specimens, confirming diagnoses of nosocomial pneumonia. Microorganism identification and antibiograms were performed using the Phoenix100 automated system from Becton Dickinson. The results revealed that 73% of the isolated microorganisms were Gram-negative bacilli, with *Acinetobacter baumannii* (29.4%) being the most common, followed by Enterobacteriaceae (28%), particularly *Klebsiella pneumoniae* (15.5%) and *Pseudomonas aeruginosa* (10.9%). Gram-positive cocci made up 22.5% of isolates, with *Staphylococcus aureus *(15.2%) being the most prevalent, while yeasts were present in 3.6% of cases. A polymicrobial nature was observed in 19.12% of samples. *A. baumannii* strains showed high resistance to most antibiotics, with an imipenem resistance rate of 88.5%; colistin was the only effective agent against these strains. In contrast, *P. aeruginosa* exhibited broad sensitivity to antibiotics, with only an 11.1% resistance rate to ceftazidime and good sensitivity to imipenem (80%). Extended-spectrum beta-lactamase production was noted in 11.5% of Enterobacteriaceae, mainly *K. pneumoniae*. Methicillin-resistant *S. aureus* prevalence was low at 11.6%, and all *S. aureus* strains were vancomycin-sensitive. The study highlights the importance of prudent antibiotic use, enhanced hospital hygiene practices, and ongoing monitoring of bacterial resistance. These measures are vital for developing therapeutic strategies suited to local epidemiology and reducing infections caused by multidrug-resistant microorganisms.

## Introduction

Nosocomial pneumonia presents a significant challenge for healthcare facilities globally, profoundly affecting the morbidity and mortality rates of hospitalized patients. Antibiotic resistance, in particular, is a critical concern that complicates the management of these respiratory infections. Our study aims to characterize the bacteriological profile of nosocomial pneumonia and evaluate the current state of antibiotic resistance. The ongoing evolution of pathogenic microorganisms and their adaptation to antimicrobial treatments highlight the importance of understanding antibiotic resistance dynamics within a hospital setting. This research employs a rigorous methodology, combining precise pathogen identification with a comprehensive assessment of their susceptibility to commonly used antibiotics. The study was conducted at the Military Hospital of Avicenne over a five-year period.

## Materials and methods

This retrospective descriptive study was conducted over five years, from January 1, 2017, to December 31, 2021, at the microbiology laboratory of the Military Hospital of Avicenne in Marrakech, Morocco. The study focused on respiratory samples, including protected distal samples (PDPs), endotracheal aspirations (ETAs), and cytobacteriological examination (CBE) of sputum. The inclusion criteria required that patients be hospitalized for at least 48 hours and have one or more positive cultures. Patients who stayed less than 48 hours or had recurrent positive cultures identifying the same pathogen during the same episode were excluded. The diagnostic samples were collected from hospitalized patients within the same hospital.

The diagnosis was based on several parameters: microscopic examination, macroscopic examination, bacterial culture and identification, and antibiogram testing. The macroscopic analysis of bacteriological samples considered their appearance and color (cloudy, purulent, or hemorrhagic). Fresh microscopic examination enabled the counting of leukocytes and epithelial cells, as well as the identification of other cells. This examination validated the deep nature of bronchial aspirations and sputum, ruling out salivary contamination based on the criteria established by Bartlett, Murray, and Washington [1] (Table 1). Additionally, Gram staining provided insights into the staining affinity of bacteria (Gram-positive or Gram-negative), their size, grouping (diplococci, clusters, chains, etc.), and the proportion of each bacterial type in polymorphic samples.

**Table 1:** Selection criteria for bronchial aspiration and sputum

Class according to Bartlett, Murray, and Washington	Epithelial cells/field	Leukocytes/field	Qualification
1	>25	<10	Rejected
2	>25	10-25	Rejected
3	>25	>25	Rejected
4	10-25	>25	Accepted

After macroscopic examination, samples were inoculated in four quadrants with a calibrated 10 µL loop on ordinary, enriched, selective, and non-selective media for culture. After incubation at 37 °C for 24-48 hours, colonies were counted. The pathogenicity threshold depends on the sample preparation method. Each bacterial type exceeding this threshold is identified and subjected to an antibiogram (Table 2).

**Table 2:** Thresholds defining pathogenicity according to sampling method CBE, cytobacteriological examination

Sampling method	Threshold defining pathogenicity
CBE of sputum	10⁷ CFU/ml
Endotracheal aspirate	10⁵ CFU/ml
Bronchoalveolar lavage	10⁴ CFU/ml
Protected specimen brush	10³ CFU/ml

Bacterial identification begins with a direct examination after Gram staining, which guides the selection of biochemical tests. Characteristics such as catalase and oxidase are sought. Definitive diagnosis is done either manually with API galleries to test biochemical properties such as sugar fermentation and nitrate reduction or automatically with the BD Phoenix™ M50 Instrument (Franklin Lakes, NJ, United States), routinely used in the HMA laboratory.

The automated liquid medium antibiogram, performed with the Phoenix™ M50 Instrument, allows for precise identification of bacterial strains and determination of their sensitivity to a wide range of antibiotics using the MIC (minimum inhibitory concentration) method. Once the bacteria are isolated and identified, the antibiogram is used to confirm the identification, assess the epidemiological spread, and determine the effective antibiotics to be reported to the clinician. Commonly used phenotypic techniques include the standard antibiogram according to the Mueller-Hinton agar diffusion method.

The list of antibiotics to be tested in the antibiogram, along with their concentrations and critical diameters, follows the recommendations of CASFM 2021 [2].

## Results

During the study period, 660 samples were processed in the microbiology laboratory for hospitalized patients. By frequency, the ICU had the highest number of cases of nosocomial pneumonia, totaling 197 cases (Table 3). The sex ratio was 5.9. Bronchoalveolar lavage was the most frequently performed examination, with a total of 278, followed by CBE of sputum with a total of 272 (Table 4). Among these samples, 251 were found positive: 115 from bronchoalveolar lavage, 72 from PDPs, and 64 from CBE of sputum (Table 4), with an increasing annual frequency from 2017 to 2020 followed by a decline in 2021.

**Table 3:** Distribution of patients according to the hospitalization department

Hospitalization department	N (%)
ICU	197 (92%)
Internal medicine	9 (4%)
Pulmonology	5 (2%)
Cardiology	1 (1%)
Ear, nose, and throat	1 (1%)
Total	213 (100%)

**Table 4:** Distribution of positive culture nosocomial pneumonia according to the type of sample CBE, cytobacteriological examination; ETA, endotracheal aspiration

Samples	N (%)	Positive culture, N (%)
Protected specimen brush	85 (12.88%)	72 (84.70%)
Bronchoalveolar lavage	278 (42.12%)	115 (41.36%)
CBE of sputum	272 (41.21%)	64 (23.52%)
ETA	25 (3.79%)	0 (0%)
Total	660 (100%)	251 (38.03%)

We observed that Gram-negative bacilli (GNB) constituted 43.9% of all isolated germs, notably characterized by the prevalence of *Acinetobacter baumannii* (29.4%) and *Pseudomonas aeruginosa *(10.9%). *Staphylococci *accounted for 18.4%, with a notable predominance of *Staphylococcus aureus *(15.2%), while *Enterococci *accounted for 28% of the isolated germs, primarily represented by *Klebsiella pneumoniae *(15.5%).

In terms of frequency, the main isolated germs were *A. baumannii*, followed by *K. pneumoniae*, *S. aureus*, and finally *P. aeruginosa*. The sex ratio for all germs combined was 5.9. This male predominance was more pronounced in patients infected with *K. pneumoniae *and *P. aeruginosa*, with sex ratios of 11, respectively.

The majority of germs were identified in the ICU, with a notable predominance of *A. baumannii *(n = 88), followed by *K. pneumoniae *(n = 46), *S. aureus *(n = 44), and *P. aeruginosa *(n = 27). The internal medicine department accounted for 11 germs responsible for pneumonia, with a frequency of 2 for *S. aureus *and *Candida albicans*, and 1 for the other identified germs. In the pulmonology department, *P. aeruginosa *ranked first with 4, followed by *K. pneumoniae *with 1. Finally, in the cardiology department, pneumonia caused by *K. pneumoniae *was recorded.

The analysis of antibiotic susceptibility from 2017 to 2021 revealed contrasting evolutions of resistance for different pathogens; isolates of *A. baumannii *showed increased resistance to the majority of tested antibiotics, although this resistance to commonly prescribed antibiotics decreased during the study period.

Regarding *P. aeruginosa *isolates, the resistance rate was 11.1% for ceftazidime, and the highest resistance rate was recorded against ticarcillin (74.2%), while strains were found to be more sensitive to amikacin (87%), gentamicin (74.2%), and imipenem (69.7%). Resistance to commonly prescribed antibiotics also decreased over the course of the study.

For *S. aureus*, the methicillin-resistant S. aureus (MRSA) rate was (11.6%). Resistance was particularly high for penicillin G (97.5%) and moderately high for erythromycin (31.3%). However, the resistance rate was lower for levofloxacin (7.32%). No strain showed resistance to vancomycin, and the resistance rate to penicillin remained virtually unchanged during the study. A decrease in resistance to cefoxitin and gentamicin was observed proportionally to an increase in sensitivity to these molecules.

For Enterobacteriaceae, 73 out of 87 isolated strains were resistant to ticarcillin, representing an 88% resistance rate. The resistance rates to cefepime, cefixime, and ceftriaxone were (49.4%), (56.7%), and (52.6%), respectively. The fluoroquinolones showed a resistance rate of 56.6% for ofloxacin and 54% for ciprofloxacin. Aminoglycosides and carbapenems showed relatively lower resistance rates (Table 5).

**Table 5:** Resistance rates to antibiotics for certain germs (in %) AMC, amoxicillin-clavulanic acid; AMK, amikacin; AZT, aztreonam; C3G, third-generation cephalosporins; CEF, cefepime; CIP, ciprofloxacin; CL, colistin; E, erythromycin; GM, gentamycin; IMI, imipenem; L, lincosamide; MRSA, methicillin-resistant *Staphylococcus aureus*; PG, penicillin G; PIP, piperacillin; PIP/TAZ, piperacillin/tazobactam; PTZ, piperacillin/tazobactam; SXT, sulfamethoxazole-trimethoprim; TIC/PIP, ticarcillin/piperacillin; VAN, vancomycin

Bacteria	PTZ	CAZ	CEF	IMI	AMK	CIP	CL	TIC/PIP	PIP	AZT	C3G	AMC	SXT	G	MRSA	PG	E	L	VAN
Acinetobacter baumannii	93.7	94.4	94	88.5	78	96.5	0						81.4	90.7					
Pseudomonas aeruginosa	22.5	21.4		21.2	13				25	11.1				25.8					
Enterobacteriaceae (all species combined)			49.4	25.3	10.7	54		88			65.7	70.6	56.5	37.3					
Klebsiella pneumoniae			60	20.8	10.9	64.4					60	63	59.6	35.6					
Escherichia coli			33.3	22.2	12.5	44.4		66.7			33.3	77.8	75	12.5					
Staphylococcus aureus													12.5	20.5	11.6	97.5	31.3	13.2	0

## Discussion

Nosocomial pneumonia remains a major concern in hospital settings, causing substantial morbidity and presenting a complex diagnostic challenge. These are lower respiratory tract infections occurring 48 hours or more after hospitalization [3], not in the incubation phase at the time of admission. We classically distinguish two types of pneumonia: ventilator-associated pneumonia, prevalent after prolonged endotracheal intubation, and non-ventilator-associated pneumonia, characterized by their occurrence after 48-72 hours of hospitalization [4]. Moreover, according to the time of onset, they are classified as early (before the fifth day) or late (after five days) [5,6].

Epidemiological data in Morocco from recent studies reveal a significant incidence of pneumonia. In particular, a study conducted at the Hassan II University Hospital in Fez in 2013 reported an incidence of 11.2%, representing 25% of infections acquired in intensive care [7]. Another investigation at the Military Hospital in Marrakech of Avicenne in 2015 reported a prevalence of 10.48%. The overall incidence varies between five and more than 20 cases per 1,000 admissions [8].

The diagnosis of pneumonia remains a challenge, relying on clinical, biological, and radiological criteria, the isolated performances of which are limited. The addition of microbiological criteria based on the analysis of respiratory samples is essential. The methods include non-invasive sampling (ETA) or invasive sampling (protected distal sampling, protected telescopic brush, bronchoalveolar lavage), requiring rapid microbiological analysis to ensure pathogen viability.

In addition to traditional methods, several microbiological alternatives have emerged, including positive blood cultures, pleural fluid cultures, suggestive histological examinations, antigenemia [9], antigenuria, serologies, as well as molecular biology techniques. New tests, such as the BioFire® FilmArray® Pneumonia Panel, multiplex PCR, exhalome analysis, and chromogenic tests, offer rapid and specific approaches [10,11].

The cultures in our study were polymicrobial in 19.13% of cases and monomicrobial in 87.13% of cases. In contrast, a study conducted in the ICU at Ibn Tofail Hospital in Marrakech highlighted the predominance of polymicrobial cultures [12].

The analysis of antibiotic sensitivity revealed variable trends in the resistance of the main pathogens.

Our study reveals a significant prevalence of GNB with an isolation rate of 73%, while Gram-positive cocci (GPC) represent 22.5%. Non-fermenting GNB are the predominant bacterial family, constituting 43.9% of the isolates, followed by Enterobacteriaceae at 28%. The most frequently isolated species are *A. baumannii *(29.4%) and *K. pneumoniae *(15.5%).

These results are consistent with the literature [13-16], highlighting the dominance of GNB, particularly non-fermenting GNB, in nosocomial pneumonia. Prevalence data from various studies confirm *A. baumannii *as the primary pathogen, generally followed by *K. pneumoniae *in the second position and *P. aeruginosa *in the third position.

*A. baumannii *resistance extends to several classes of antibiotics, including broad-spectrum beta-lactams, aminoglycosides, and fluoroquinolones. The resistance rate to the piperacillin-tazobactam combination exceeds 75%, approaching our series where it reaches 93.75%. However, significant variations are observed in different regions, with a reported resistance rate of 37.8% in Jordan and 100% in Turkey [12,17].

In our series, resistance to third-generation cephalosporins reaches 94.4%. This percentage aligns with those reported in certain previous studies [12,17]. Regarding imipenem, the resistance was 88.5%, which aligns with Chinese data reporting a resistance of 88.6%. Notably, this resistance showed a downward trend over the course of our study period, decreasing from 100% in 2017 to 88.5% in 2021 [18].

According to a study conducted in Marrakech, *P. aeruginosa *resistance to ceftazidime is 0%, while resistance to imipenem is 5.3%. These results are similar to our study, where *P. aeruginosa *remains relatively sensitive to these two molecules [12]. The highest resistance rate in our series concerns gentamicin, with 25.8% resistance, correlating with a previous study by Bahçe et al. [16]. The majority of other antibiotics remain effective against our strains, compared to other studies.

For all Enterobacteriaceae, the resistance rates to beta-lactams fall within similar ranges as those reported by other studies [12,19]. For *K. pneumoniae*, resistance rates remain within comparable ranges for the majority of antibiotics tested. Regarding *Escherichia coli*, our strains exhibit lower resistance to cephalosporins and gentamicin compared to international data [7,15,20].

In our series, low resistance was observed toward linezolid and cotrimoxazole in *S. aureus*, particularly compared to international studies. Resistance to penicillin G was very prevalent, affecting 97.5% of our strains, consistent with the results of other cited studies. The rate of MRSA was 11.6%, consistent with the findings of Golia et al. [20], although higher rates have been reported in other studies [21,22]. Vancomycin remains effective against all strains of *S. aureus*, correlating data from the literature.

The limitations of our research include a potentially limited and non-diverse sample size, as well as variability due to manual techniques and laboratory conditions. The automated Phoenix® M50 system may not detect all bacterial resistance mechanisms, and the range of antibiotics tested might exclude newer or less common options.

## Conclusions

Nosocomial pneumonia represents a major public health issue as the second most common infection in hospital settings. Despite diagnostic and therapeutic advancements, the prognosis of these infections remains pessimistic. The diagnosis of nosocomial pneumonia is based on clinical, biological, radiological, and bacteriological criteria. Our retrospective study revealed a predominance of GNB (73%), with *A. baumannii *(29.4%), *K. pneumoniae *(15.5%), and *P. aeruginosa *(10.9%) as the main pathogens. GPC include *S. aureus *(15.2%) and *Streptococcus pneumoniae *(2.3%), with 19.13% of positive samples being polymicrobial. The increasing rates of antibiotic resistance complicate patient management, underscoring the necessity for rational prescriptions based on antibiogram results. Regular surveillance of antibiotic resistance at healthcare facilities is crucial for defining appropriate therapeutic strategies. The implementation of effective preventive measures requires an integrated program across all hospital services.
